# Neutron diffraction measurements of weld residual stresses in three-pass slot weld (Alloy 600/82) and assessment of the measurement uncertainty

**DOI:** 10.1107/S1600576720009140

**Published:** 2020-08-14

**Authors:** Vasileios Akrivos, Robert C. Wimpory, Michael Hofmann, Beverly Stewart, Ondrej Muransky, Mike C. Smith, John Bouchard

**Affiliations:** a Department of Mechanical, Aerospace and Civil Engineering, School of Engineering, The University of Manchester, Sackville Street, Manchester M13 9PL, UK; b Helmholtz-Zentrum Berlin für Materialien und Energie, Hahn-Meitner-Platz 1, Berlin 14109, Germany; c Forschungs-Neutronenquelle Heinz Maier-Leibnitz (FRM II), Technische Universität München, Lichtenberg Strasse 1, Garching, München D-5747, Germany; d Materials Engineering, The Open University, Walton Hall, Milton Keynes MK7 6AA, UK; e Australian Nuclear Science and Technology Organisation (ANSTO), Lucas Heights, NSW 2234, Australia; f School of Mechanical and Manufacturing Engineering, University of New South Wales Sydney, Kensington, NSW 2052, Australia

**Keywords:** neutron diffraction, residual strain, welding dilution, uncertainty, Bayesian mean

## Abstract

This paper describes in detail two neutron diffraction residual stress measurements, performed on the ENGIN-X neutron scattering instrument at the ISIS facility in the UK and on the SALSA instrument at the Institut Laue–Langevin in Grenoble, France. The measurements were conducted as part of the NeT Task Group 6 (TG6) international measurement round robin on an Alloy 600/82 multi-pass weldment – a slot in an Alloy 600 plate filled with three Alloy 82 weld beads, simulating a repair weld. This alloy/weld combination is considered challenging to measure, due to the large grain size and texture in the weld, and large gradients in the stress-free lattice parameter between the parent and weld metal. The basic principles of the neutron diffraction technique are introduced and issues affecting the reliability of residual stress characterization are highlighted. Two different analysis strategies are used for estimation of residual stresses from the raw data. Chemical composition studies are used to measure the mixing of parent and weld metal and highlight the steep lattice parameter gradients that arise as a consequence. The inferred residual stresses are then compared with three sets of measurements performed on the same plate by other NeT partners on E3 at the HZB in Berlin, STRESS-SPEC at the FRM II in Munich and KOWARI in Sydney. A robust Bayesian estimation average is calculated from the combined five-instrument data set, allowing reliable best estimates of the residual stress distribution in the vicinity of the weldment. The systematic uncertainties associated with the residual stress measurements are determined separately in the weld and parent materials, and compared with those in the NeT TG4 benchmark. This is a three-pass slot-welded plate fabricated from American Iron and Steel Institute AISI 316L(N) austenitic stainless steel, and is normally considered less challenging to measure using diffraction techniques than all nickel welds. The uncertainties in the stress measurements by neutron diffraction for these two weldments seem to be comparable.

## Abstract 2

Residual strain measurements were conducted on an Alloy 600/82 weld on five diffractometers, and two different approaches were used to analyse the data and estimate the stresses. The large body of data enabled estimation of the Bayesian mean of the residual stress distribution and a statistically robust estimation of the residual stress uncertainty associated with the method.

## 1. Introduction   

Residual stresses within a component can adversely affect its structural integrity and reduce its lifetime, as it becomes more susceptible to degradation mechanisms such as stress corrosion cracking, fatigue and creep (Bouchard, 2001 ▸). Weld residual stresses develop due to shape misfits that arise from the thermal and mechanical loads applied simultaneously during welding temperature cycles. They can exceed the yield strength of the material, and in the absence of post-weld heat treatment, these high stresses remain in welded components as they enter service. Nickel alloys are widely used in welded components in pressurized water reactors (PWRs). Examples are steam generator tubing and divider plates, and the dissimilar metal welds connecting components made of low alloy steel to those made from austenitic steel (Ballinger, 2008 ▸; Clement, 2008 ▸). Nickel alloy dissimilar metal welds in PWRs are not heat treated, so contain significant residual stresses. When nickel Alloy 82 or 182 welding consumables are used, the interaction between material, residual stress (RS) and circuit water chemistry can lead to primary water stress corrosion cracking (IAEA, 2011 ▸). Therefore, substantial efforts have been made to understand RS development in nickel alloy dissimilar metal welds, via both measurement and simulation (White *et al.*, 2007 ▸; Lippold *et al.*, 2009 ▸; Norring & Engström, 2008 ▸; Bruemmer *et al.*, 1999 ▸; Marlette *et al.*, 2010 ▸; Brust *et al.*, 2010 ▸; Fredette *et al.*, 2011 ▸; Shim *et al.*, 2010 ▸; Rathbun *et al.*, 2011 ▸; Smith, Muransky, Bendeich & Edwards, 2010 ▸; Smith, Muransky, Goodfellow *et al.*, 2010 ▸). These have highlighted the need both to make RS measurements using multiple techniques with different characteristic errors and to interpret simulation predictions in the light of reliable measured estimates of RS. This approach is mandated in the R6 structural integrity assessment procedure in the UK (EDF, 2015 ▸; Bate & Smith, 2016 ▸).

The mission of the European Network on Neutron Techniques Standardization for Structural Integrity (NeT) is to develop experimental and numerical techniques and standards for the reliable characterization of residual stresses in structural welds. NeT was first established in 2002, and involves over 35 organizations from Europe and beyond. It operates on a ‘contribution in kind’ basis from industrial, academic and research facility partners. Each problem examined by the network is tackled by creating a dedicated Task Group, which undertakes measurement and modelling studies and the interpretation of the results. NeT Task Group 6 (TG6) was started in early 2012 and examines the behaviour of an Alloy 600 plate containing a three-pass slot weld, made using the tungsten–inert-gas (TIG) welding process with an Alloy 82 filler. TG6 is a natural follow-on from the NeT TG4 project, a three-pass slot weld in AISI 316L(N) steel (Smith *et al.*, 2018 ▸). The RS measurement campaign in NeT TG4 was the most extensive ever undertaken on a weldment benchmark, and its results allowed detailed insight into the real-world reliability of diverse RS measurement techniques when applied to welds in an austentic stainless steel. RS measurements using diffraction techniques in nickel alloy weld metals are normally considered more challenging than those in AISI 316, because of the nickel alloys’ tendency to develop large grain sizes. Net TG6 thus offers the opportunity to address this issue in the context of a large international research project, where repeat diffraction-based measurements are made at multiple facilities around the world, and are then combined with measurements made using strain-relief methods. In the past a substantial effort has been made by most of the residual stress neutron facilities in the world gathering strain data on a shrink-fit ring and plug round robin sample as part of the Versailles Project on Advanced Materials and Standards (VAMAS) project (Daymond *et al.*, 2002 ▸).

The current study examines the neutron diffraction measurements made in the NeT TG6 benchmark weldment. Residual stresses have been determined using the time-of-flight diffractometer ENGIN-X (ISIS, Didcot, UK) and the monochromatic neutron diffractometer SALSA (ILL, Grenoble, France). The results were supplemented by three more sets of measurements performed using monochromatic neutron diffractometers, namely E3 (HZB, Berlin, Germany), STRESS-SPEC (FRM II, Munich, Germany) and KOWARI (ANSTO, Sydney, Australia). An overview is given of the methods of post processing the data. A comprehensive presentation of the stress distribution is given through the different line plots, with an emphasis on the systematic stress uncertainties of the measurements on this specimen and how they compare with the TG4 benchmark (Smith *et al.*, 2018 ▸) made of 316L(N) stainless steel.

## 2. NeT TG6 benchmark specimens   

The NeT TG6 specimen is a three-pass slot weld in Alloy 600 (also referred as Inconel 600) Ni–Cr alloy, made using the TIG welding process with Alloy 82 filler. Each TG6 specimen consists of a plate with a central groove filled with three superimposed weld beads. The nominal dimensions of the plate are 200 × 150 × 12 mm, while the slot is 76 mm long and 5 mm deep. This provides significant structural restraint while remaining thin enough to ensure that diffraction measurements of residual stresses are still feasible.

**Figure 1:**
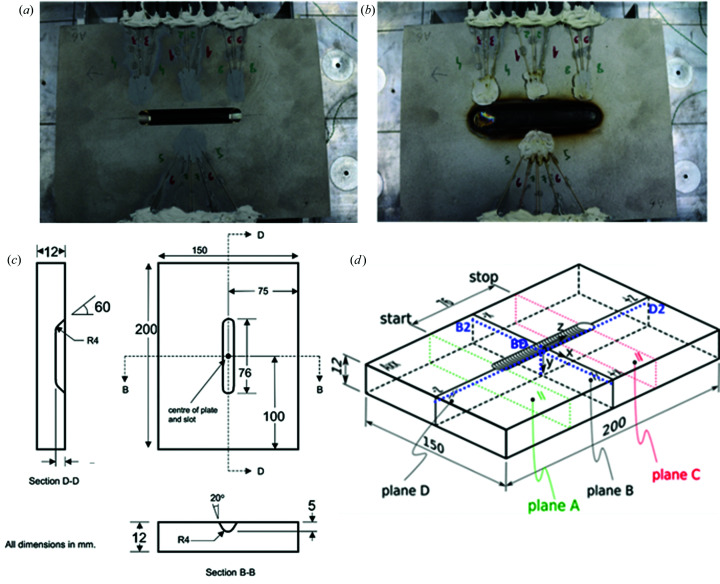
Specimen A6 instrumented with thermocouples (*a*) before and (*b*) after welding. (*c*) A schematic representation of the TG6 specimen showing the dimensions of the plate and machined slot, and (*d*) the coordinate system. Start and stop refer to the start positions and the measured lines (Smith *et al.*, 2014 ▸).

A sketch of the specimen, illustrating also the origin of the coordinate system, is presented in Fig. 1 ▸, which also depicts the measurement planes B and D. Plane D goes through the entire specimen at the middle of the slot parallel to the welding direction and plane B goes through the entire specimen at the middle of the slot transverse to the welding direction. The B and D measurement lines are parallel to the top surface in their respective planes. The line with the highest priority is line BD which sits at the intersection of planes D and B and goes through all three weld beads plus the thermo-mechanically cycled parent material underneath the weld.

The chemical compositions and basic tensile properties of both the base and filler materials are presented in Table 1 ▸. All the TG6 specimens were manufactured at the EDF laboratory in Chatou, France, using an automated robotic TIG welding machine to ensure repeatability. The TG6 specimen used in the diffraction measurements campaign was labelled A5. Another specimen labelled A6 was used for an exploratory contour-method RS measurement and then cut into pieces and used for characterization studies and extraction of stress-free reference (*d*_0_) samples for neutron diffraction measurements (see Section 3.4).

**Figure 2:**
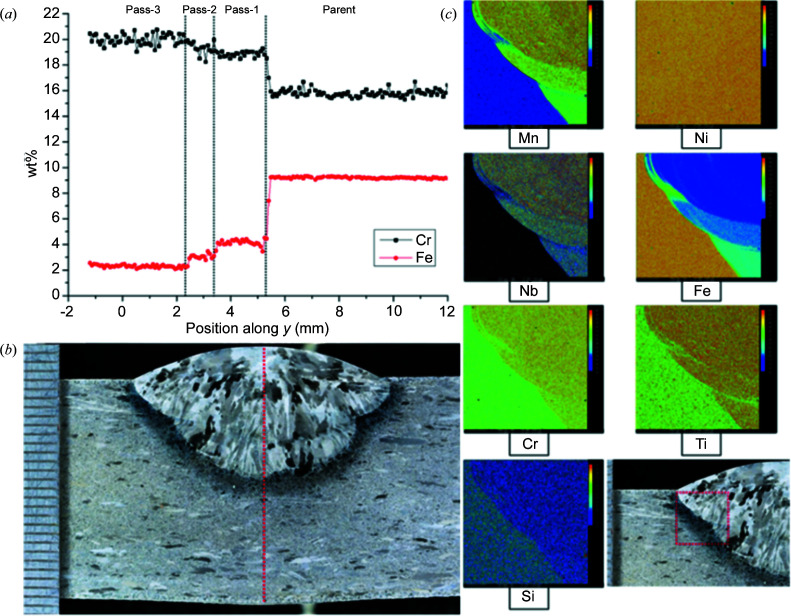
(*a*) Chemical composition BD line scans on a cross section taken at the weld mid-length of the A6 three-pass sample. (*b*) The optical macrograph as revealed after etching. (*c*) The 2.8 × 2.8 mm element distribution maps of the A6 three-pass sample. Note that the element concentration scale varies for different element distribution maps.

Studies were conducted to verify the chemical compositions of both the parent material and the three weld beads, all of which had slightly different compositions due to the differing amounts of dilution of each successive weld pool with melted parent material and re-melted weld beads. For this purpose, electron probe micro-analysis (EPMA) and a Cameca SX100 electron microprobe were employed to obtain both quantitative elemental analyses and point measurements via wavelength-dispersive spectroscopy. A series of point scans along lines with a 75 µm step size were made, passing though the weld in the through-thickness direction. The line plots depict the dilution effects quantitatively with regard to iron (Fe) and chromium (Cr) content [Fig. 2 ▸(*a*)]. Within the fusion zone the Fe content decreases whilst the Cr content increases after the deposition of a subsequent pass, as can be seen by comparison with the optical macrograph [Fig. 2 ▸(*b*)]. Element distribution maps were also captured at regions near the fusion boundary for, in addition to Fe and Cr, manganese (Mn), nickel (Ni), niobium (Nb), titanium (Ti) and silicon (Si). The colour-coded maps are only for qualitative analysis. The scale of each element map is in units of counts per second and varies between maps [Fig. 2 ▸(*c*)].

Grain-size studies have revealed a bi-modal grain structure in the parent material with a few large grains (∼500 µm) within a matrix of small grains (∼20 µm), and very large columnar grains, of the order of a millimetre, developed in the weld fusion zone (Akrivos & Smith, 2019 ▸). Texture measurements, carried out at ANSTO in Australia (Ohms *et al.*, 2015 ▸) using a trial three-pass weldment, confirmed a weak rolling texture in the parent material and a relatively weak cube texture at the bottom of the weld in the area of the first deposited pass. However, a strong cube texture was revealed at the top of the weld where the final weld bead is located.

## 3. Neutron diffraction measurement of weld residual stresses   

### 3.1. Theory   

The RS measurement campaign was performed using the neutron diffraction (ND) technique which is based on Bragg’s law (Bragg, 1912 ▸), 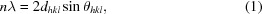
where *n* is an integer, λ is the neutron wavelength, *d* is the lattice spacing or the distance between sets of parallel crystallographic planes characterized by the Miller indices *hkl*, and 2θ is the scattering angle. Effectively, in polycrystalline materials the accurate measurement of the distance between similarly oriented planes of atoms using the crystal lattice can be used as a tool to measure elastic strain. Every change in the lattice spacing (Δ*d*) denotes a residual strain through the following equation: 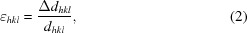
which is derived by measuring in both the stressed (*d*) and the unstressed condition (*d*_0_). In cases of fitting several peaks the strain can be evaluated by calculating the lattice parameter *a*, which is related to the lattice spacing as follows: 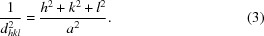
Thus for strain calculations equation (2) becomes
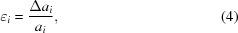
where *a_i_* denotes either *a* or *a*_0_, which are the lattice parameter of the material in the strained and unstrained condition, respectively. Once the three principal strain components are measured, the stress in each direction is calculated using Hooke’s law through the following equation: 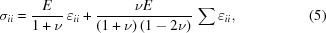
where *E* is the elastic modulus and ν is Poisson’s ratio. A bulk or engineering *E* is used for time-of-flight instruments, and crystallographic moduli relevant to the single planes (*i.e.* 311) being measured are used for monochromatic instruments. The macroscopic and crystallographic values of *E* and ν used in this study were the same (*E* = 206 GPa, ν = 0.29). They were sourced from the article by Holden *et al.* (1998 ▸) and provided by the NeT protocol (Ohms *et al.*, 2015 ▸), taking into account the material’s main composition elements (74 wt% Ni, 14 wt% Cr). This set of values was used both for parent and weld metal, although the constants are expected to be slightly different and some anisotropy is expected in the weld metal.

### 3.2. Time-of-flight measurements of weld residual stresses   

**Figure 3:**
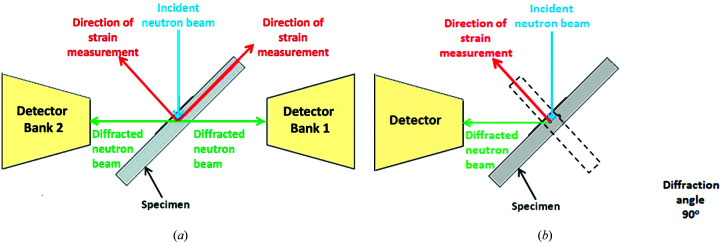
(*a*) The neutron diffraction configuration on ENGIN-X, with the two detectors acquiring two strain components simultaneously. (*b*) The same configuration at SALSA with one detector. The orientations of the specimen are necessary to measure the two strain components.

One set of measurements was performed on the dedicated engineering diffractometer ENGIN-X at the ISIS facility of the Rutherford Appleton Laboratory (UK). At the ISIS neutron spallation source, the measured parameter (while keeping the diffraction angle constant) is the time of flight of the neutrons, which is directly related to their speed and wavelength. Only one measurement is required for information about several reflection planes to be captured (Withers & Bhadeshia, 2001 ▸). Two orthogonal strain components are measured simultaneously via two opposing detector banks positioned at a Bragg angle (2θ) of 90°, as seen in Fig. 3 ▸(*a*). Thus for a constant angle (θ), a change in time of flight (Δ*t*) can be used to measure strain. A gauge volume of 2 × 2 × 2 mm was used for the measurements along plane D and of 3 × 2 × 2 mm for the measurements on plane B. No angular oscillation was applied in this experiment. ENGIN-X benefits from detectors with large angle coverage both horizontally and vertically, and hence is less susceptible to grain-size issues as it averages over more grains.

The pulse of neutrons used in the time-of-flight setup consists of a full spectrum with many different wavelengths; hence a full diffraction pattern can be analysed at a constant angle. Peak fitting of the diffraction pattern is performed by a Pawley refinement (Pawley, 1981 ▸). The predicted lattice parameter is a result of a least-squares fit to selected peaks of that spectrum. The acquired full diffraction spectra were analysed using the *Open Genie* data reduction and analysis software (Akeroyd *et al.*, 2002 ▸) which employs the Rietveld refinement code *GSAS* (Von Dreele *et al.*, 1982 ▸) and provides a full-pattern refinement of the diffraction spectra via a library of common engineering materials. The peak intensities are unconstrained to account for materials with texture (Pawley, 1981 ▸). To confirm the convergence of the refinement, preliminary fits of the lattice parameters are acquired by refinement of the most intense peak (Santisteban *et al.*, 2006 ▸). Finally, a full list of lattice parameters in the weldment and stress-free samples was calculated and used to infer the associated stresses.

### 3.3. Constant-wavelength measurements of weld residual stresses   

A second measurement campaign was conducted on the SALSA instrument at the ILL (Pirling *et al.*, 2006*b* ▸). This uses a high continuous flux of monochromatic neutrons to determine the lattice parameters (Acevedo *et al.*, 2012 ▸). The diffraction peaks at particular 2θ angles are measured as a result of the neutrons diffracting in the material (Withers, 2007 ▸). The wavelength was fixed to λ ≃ 1.57 Å, which allows measurement of the peak from the (311) planes at a scattering angle 2θ close to 90°. This set of planes was chosen since they are known to be less susceptible to intergranular stresses, thus better resembling the bulk stresses in the material (Drezet *et al.*, 2012 ▸). The configuration of this experiment can be found in Fig. 3 ▸(*b*).

A 2 × 2 × 2 mm gauge volume was used on all measurements to ensure a relatively strong detected signal of diffracted neutrons. Due to the coarse-grained nature of the weld material, a ±5° angular oscillation was also used to increase the number of diffracting grains and the average of the peaks was recorded at each point. A threshold value was defined on the counter to ensure roughly the same peak statistics. The diffracted intensities, *I*, obtained from the detector were analysed and integrated using the *LAMP* software calibrated for the beamline (Pirling *et al.*, 2011 ▸). A background noise correction was performed on each of the unidirectional diffraction peaks obtained and a Gaussian fit was then applied to infer the 2θ position. The uncertainty was provided as a fitting error in each measurement by the *LAMP* software. There is an additional positioning uncertainty of 20–30 µm that is associated with sample to mounting plate and plate to hexapod fixture, which was identified in a previous study using the SALSA instrument (Pirling *et al.*, 2006*a* ▸). However, this was neglected in the current analysis as the main source of error was identified to arise from the relative positioning/correlation of the stress-free reference and plate specimens, which both have a chemical composition gradient between the parent and weld metal that could introduce significant systematic uncertainties in strain determination. An example of the steep chemical compositional gradients is presented in Fig. 5, showing the through-thickness-of-plate 2θ_0_ gradients at the weld centre line as measured in one of the extracted short pins described below. An ∼1 mm distortion was taken into account for measurement points in plane B. No distortion was taken into account for line measurements along plane D.

### 3.4. Analysis of neutron diffraction data   

#### 3.4.1. Use of *d*_0_ pins   

**Figure 4:**
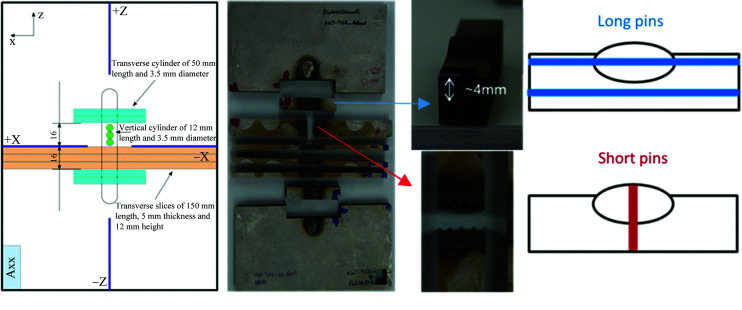
(*a*) The cutting plan for extraction of reference specimens from the A6, TG6 specimen (Ohms *et al.*, 2015 ▸). (*b*) The A6 specimen after extraction of the stress-free pins and slices. (*c*) The exact locations of the extracted pins used as stress-free reference samples.

The specimen labelled A6 (Fig. 4 ▸) was used for an exploratory contour method RS measurement, and then cut into pieces and used for characterization studies and extraction of stress-free reference (*d*_0_) samples for ND measurements [Fig. 4 ▸(*b*)]. These take the form of 3.5 mm diameter pins, circumferentially notched at intervals along their length, extracted from locations representative of measurements on a through-wall line (short pins) and measurements made on transverse lines (long pins). Four pins accompanied specimen A5 to all the instruments. The two 50 mm ‘long’ pins had their axes aligned transverse to the welding direction. One had been extracted at a depth of ∼4 mm and passed through the parent material, heat-affected zone (HAZ), weld metal, HAZ and parent material again at this depth, while the second, extracted at a depth of ∼9 mm, only passed through parent material. The two 14 mm ‘short’ pins were extracted close to the weld mid-length on the weld centre line, with their axes aligned with the through-thickness direction going from the top of the weld cap to the bottom surface of the plate [Fig. 4 ▸(*c*)]. These passed through all three weld passes, the HAZ and the remaining parent material ligament.

The main challenges for strain measurements in the TG6 specimen come from the combination of a deformed specimen, the differences in chemical compositions of the parent and weld materials and consequently dilution of parent into weld that causes large *a*_0_ gradients at material interfaces, and offsets between the transverse pin extraction depth and the depths of measurement lines.

**Figure 5:**
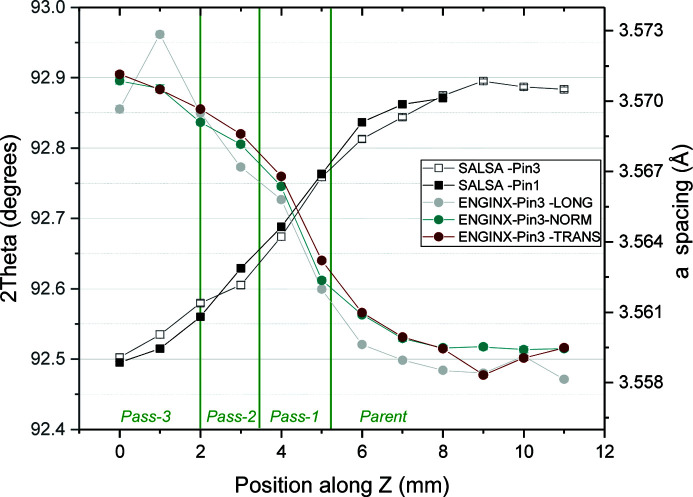
The 2θ and *a* spacing values measured through the specimen thickness in the stress-free condition using the extracted pins on the SALSA diffractometer. The error bars are within the size of the symbols.

Large *a*_0_ gradients through the weld thickness and close to the fusion zone were measured, as can be seen in Fig. 5 ▸. The steep chemical compositional gradients mentioned before cause *a*_0_/2θ_0_ gradients through the thickness of the plate at the weld centre line as measured in one of the extracted short pins. Therefore, it was critical that the stress-free lattice parameter measurements made in pins extracted from specimen A6 correspond to the exact locations of the strain measurements made in specimen A5, assuming both welds are nominally the same.

#### 3.4.2. Position fitting for ENGIN-X data   

**Figure 6:**
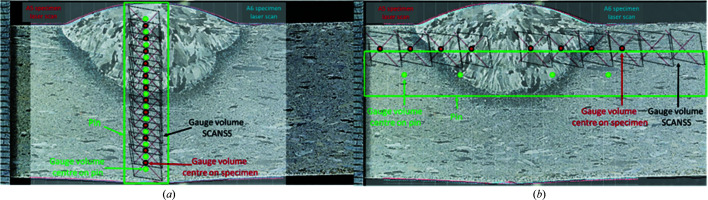
Metallography on transverse cross section at weld mid-length superimposed on laser scans of specimens A5 and A6 plus the *SCANSS* model of measurement points on (*a*) the BD line and (*b*) line B2. The misalignment is illustrated in both the *x* and *y* directions between the measurement points on the weldment and measurement points on the stress-free pins.

The deformed shapes of both specimens after welding were measured using a hand-held laser scanner. It was found that the surface profiles on plane B at the weld mid-length were almost identical in both A5 and A6. Both specimens contained a convex weld crown, and both showed bulging of the bottom surface in the ligament beneath the weld due to plastic deformation during welding. These features meant that neither end of the vertical pin extracted from this region could be assumed to be at the undeformed surface of the specimen. The model for each measurement line that was created using the *SSCANSS* software (James *et al.*, 2004 ▸) was then superimposed on the laser-scanned profiles to illustrate the exact locations of the gauge volume at each measurement point. Additionally, for each set of profiles, macrographs were available for comparison. The laser scans at the weld mid-length in the transverse direction were placed on top of the metallography of the same location for lines BD [Fig. 6 ▸(*a*)] and B2 [Fig. 6 ▸(*b*)].

**Figure 7:**
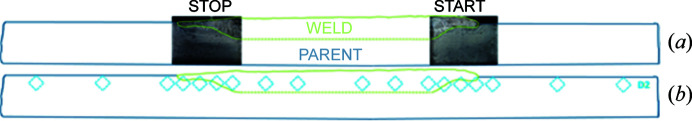
(*a*) The longitudinal profiles of specimens A5 and A6 on plane D, superimposed upon the metallography of the weld start and stop ends. The fusion boundary on plane D is inferred from the metallography. The green dashed line is the suggested fusion boundary from the metallography available. (*b*) The *SCANSS* model superimposed on the laser-scanned profiles and the inferred fusion boundary to locate measurement points on line D2.

Likewise, the macrographs at the start and stop ends in the longitudinal direction were used for line D2 which sits 2 mm below the top surface (Fig. 7 ▸). An ∼0.5 mm offset was identified between the BD measurement points and the *a*_0_ values of the ‘short’ pins [Fig. 6 ▸(*a*)]. The BD *a*_0_ curves were shifted by that amount in order to match the measurement locations in the plate. As for the transverse long pin, the exact locations where the reference values were acquired are presented in Fig. 6 ▸(*a*), showing a significant misalignment with the actual strain measurement positions.

The position fitting process employed for lines B2 and D2 enabled the estimation of a parent–weld fraction within the gauge volume at locations close to the fusion boundary. Two sets of *a*_0_ values were used, one for the weld and one for the parent. Each set consisted of three *a*_0_ values, one for each direction, accounting also for detection bank consistency. For each line the curves of *a*_0_ were also based on a linear rule of mixtures. The *a*_0_ values of points sitting on the parent–weld intermediate zone were calculated as the fractions of the parent and weld *a*_0_. This technique was implemented on line B2 and D2 measurements that go through the weld.

#### 3.4.3. The zero normal stress assumption   

A biaxial stress field was assumed, taking advantage of the low thickness of the plate. This involved the calculation of a set of θ_0_ values that effectively assume the stresses in the normal direction to be zero. Indeed, the biaxiality in stresses was implemented by zeroing the stress component in the normal direction,
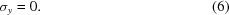
This equation can be also written in the form
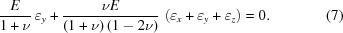
Then, one can solve for ∊_*y*_,
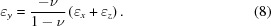
The strain in the normal direction can also be written in terms of the scattering angle using Bragg’s law [equation (1)] and equation (4),
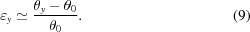
Finally, by combining equations (8) and (9) and solving for θ_0_,
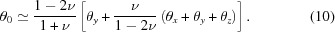
It should be noted that the plane stress assumption still involves some uncertainty. The normal stresses within the plate are relatively low but not zero. Preliminary numerical simulations performed and presented in a previous study (Smith *et al.*, 2016 ▸) also justify this. The normal stress predictions were in the region of ±30 MPa located in the weld and the weld–parent interface. Previous studies on the TG4 plate (Moturu, 2015 ▸; Smith *et al.*, 2015 ▸) which is 50% thicker (18 mm instead of 12 mm) revealed the normal stresses to vary within ±60 MPa.

### 3.5. Validation of weld residual stress measurements   

Additional strain measurements have been performed on the same welded specimen using the same stress-free samples by other members of the NeT network. Specimen A5 has been measured on three different instruments at reactor neutron sources, namely E3 at HZB in Berlin, STRESS-SPEC at FRM II in Munich (Wimpory *et al.*, 2018 ▸) and KOWARI at ANSTO in Sydney. The measured residual stresses obtained on E3, STRESS-SPEC and KOWARI have been kindly provided to the present authors for comparison purposes. The STRESS-SPEC and E3 measurements were performed on line BD and were fitted using stress-free reference data from the measured short pins. The STRESS-SPEC measurements also include lines B2 and D2, which were fitted using the zero normal stress assumption. The KOWARI measurements were fitted using the zero normal stress assumption. Table 2 ▸ summarizes the lines measured in each diffractometer and the assumption employed to calculate the stresses in the three orthogonal directions.

A robust Bayesian estimation (RBE) average of the data using the ‘duff data’ approach (Sivia, 1996 ▸) was calculated. This was done for each of the three orthogonal directions for each stress position (Sivia, 1996 ▸; Daymond *et al.*, 2002 ▸). This analysis is in general less susceptible to outliers than a conventional mean value and has been employed already for the NeT TG1 data (Wimpory *et al.*, 2009 ▸). The RBE technique involves the input of quoted uncertainties corresponding to each stress value. These uncertainties, however, are often underestimated in neutron RS measurements and the average can also be biased towards values with the lowest quoted uncertainties. Nevertheless, the spread of the residual values (after subtracting the RBE average from each data set) can give a great insight into the accuracy of each data set, as well as indicating whether the original quoted uncertainties were appropriate or not. The newly calculated uncertainties can be used to recalculate the RBE, *i.e.* feeding back the values.

**Figure 8:**
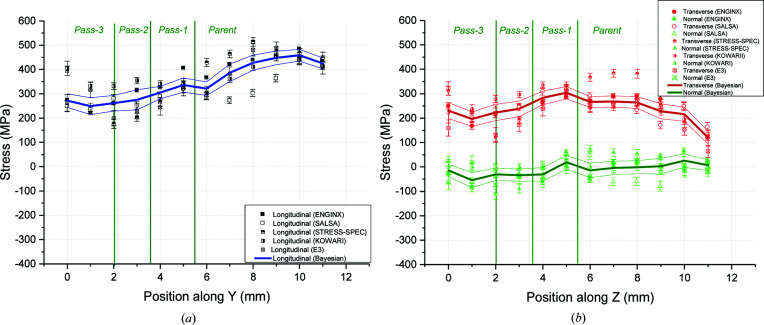
Stresses measured and the RBE for line BD along (*a*) the longitudinal and (*b*) the transverse and normal orthogonal directions.

Calculating a standard deviation of the residual values of each data set (after subtracting the RBE average) can also be subject to outliers. In order to avoid this, the standard deviations were calculated using residual fits (or *R*-fits) (Wimpory *et al.*, 2009 ▸) as well as in the conventional way. These *R*-fits give a value that is closer to the true underlying random uncertainty. With *R*-fits the residuals (on the *y* axis or ordinate) are arranged in magnitude order equidistantly on an *x*-axis (abscissa) scale from −100 to 100%, and a linear fit between −68.27 and 68.27% is applied (corresponding to ±1 SD, *i.e.* one standard deviation). The gradient from the fit, when multiplied by 68.27, provides a value of the standard deviation (random uncertainty) of the data set that is less susceptible to the influence of outliers, which are in the ±68.27–100% region. The constant value of the linear fit also provides a systematic uncertainty value which is also less susceptible to outliers. The RBE means were calculated individually for each line based on the five sets of raw data available. The means for lines BD, B2 and D2 are presented in Figs. 8 ▸–10 ▸ ▸, respectively.

## 4. Results   

### 4.1. Residual stresses along the BD line   

Line BD has been assessed as the most important set of measurement points since it passes through all the different zones with different thermo-mechanical histories. It is located at the weld mid-length where the weld heat source (*i.e.* welding torch) has achieved stable welding conditions [Fig. 6 ▸(*a*)]. It assesses the RS profile through the thickness of the weldment starting from the top surface and measuring the RSs in all the weld beads, thus sampling more weld metal which is always more challenging to measure due to the large grain size and texture. The five sets of data available enabled a more robust evaluation of the uncertainty associated with the results. Moving further down towards the bottom surface, measurements are then captured in the HAZ and the cyclically hardened parent zone. Hence, the measurement line goes through non-uniform thermal cyclic deformation zones. Fig. 8 ▸ shows the stresses measured on ENGIN-X, SALSA, STRESS-SPEC, E3 (Wimpory *et al.*, 2018 ▸) and KOWARI (Muransky, 2016 ▸).

Both longitudinal and transverse RSs are tensile in the BD line. The longitudinal stress is lower in the weld than in the parent due to the different mechanical properties between the weld and parent metal. Peak longitudinal stresses are found close to the bottom surface of the plate where the parent material has plastically deformed more. The transverse stress profile is somewhat different, with peak stresses located around the first pass of weld metal decreasing to almost zero at the bottom surface of the plate. However, the two stress components are fairly similar in distribution and magnitude within the fusion zone. The normal RSs appear to be very low and about ±50 MPa. A small compressive stress field is located in the fusion zone region, which was also predicted in the preliminary finit element (FE) results (Smith *et al.*, 2016 ▸). This is expected to have an effect on the other two stress components inferred from the KOWARI set of data which assumed zero normal stresses for this line.

### 4.2. Residual stresses along the B2 line   

**Figure 9:**
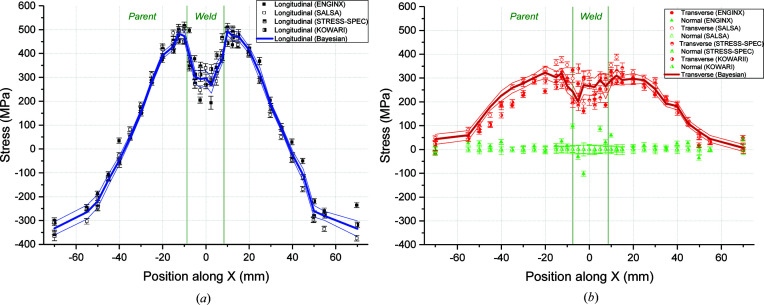
Stresses measured and the RBE for line B2 along (*a*) the longitudinal and (*b*) the transverse and normal orthogonal directions.

Line B2 is 2 mm below the top surface at weld mid-length in the transverse direction. It passes through the weld very close to the boundary between pass-2 and pass-3, and the coarse-grained heat-affected zone (CGHAZ) on both sides of the weld. The RSs calculated using measurements from four instruments are presented in Fig. 9 ▸. Longitudinal stresses [Fig. 9 ▸(*a*)] start as highly compressive (∼ −300 MPa) near the edge of the plate and gradually increase and turn into highly tensile (450–500 MPa) covering the deformed parent material next to the fusion zone (*x* = −20 to −10 mm and 10 to 20 mm). The longitudinal stress drops within the weld metal to about 300 MPa.

Transverse tensile stresses of up to 300–320 MPa have developed in the area close to the fusion zone and decrease gradually further away. The transverse stresses drop to 200–230 MPa in the weld metal and follow an M-shaped profile within the fusion zone [Fig. 9 ▸(*b*)]. The deviation in the determined longitudinal stresses within the parent material and the fusion zone, fitted by the same methodology, is about 50 MPa, which is also another depiction of the true uncertainty in the measurements.

### 4.3. Residual stresses along the D2 line   

**Figure 10:**
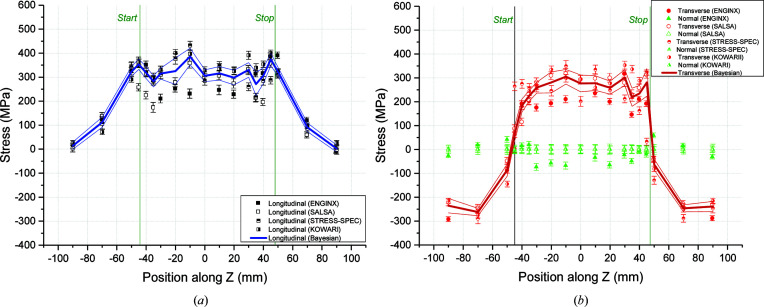
Stresses measured and the RBE for line D2 along (*a*) the longitudinal and (*b*) the transverse and normal orthogonal directions.

Line D2 is located 2 mm beneath the top surface and passes through pass-2 and pass-3 weld material. The different sets of stress obtained from the measurements calibrated either by position fitting or by using the the zero normal stress approach are presented in Fig. 10 ▸. A peak in the tensile longitudinal stress of about 380 MPa is seen in the plastically deformed parent zone (*z* = −50 to −40 mm and 45 to 50 mm) [Fig. 10 ▸(*a*)]. The longitudinal stresses then decrease to zero at the ends of the plate, as expected. Stresses fall by 70–100 MPa as the CGHAZ is approached (*z* = −45 to −40 mm and 40 to 45 mm) and similar stresses are observed within the fusion zone (*z* = −35 to 35 mm). Transverse compressive stresses (−250 to −300 MPa) are seen at the ends of the plate [Fig. 10 ▸(*b*)]. They turn into tension as the welded area is approached and along the fusion zone. The tensile transverse stresses close to and within the fusion zone are balanced by compressive stresses of about −250 to −300 MPa at the ends of the plate.

The consistency between the Bayesian means of the stresses was evaluated for the measurement point (0, 0, 2) at the weld centre line and weld mid-length, located 2 mm below the top surface. This point is common to lines BD, B2 and D2. The longitudinal stress at that location was 260 MPa in line BD but 300 MPa in lines B2 and D2. The transverse stresses were found to be 220, 270 and 280 MPa in lines BD, D2 and B2, respectively. The lower values for both stress components found on line BD compared with lines B2 and D2 are attributed to the different analysis strategy followed. For line BD, all sets of data were analysed using the *d*_0_ pins, apart from the KOWARI set which was analysed using the zero normal assumption. In contrast, for lines B2 and D2 all sets of data were inferred by adopting the zero normal stress assumption, except for the ENGIN-X set of data that made use of the position-fitting approach. Preliminary FE simulations predicted a small compressive stress in the fusion zone of about 30 MPa. Hence, the sets that adopted the zero normal stress approach are expected to produce a slightly higher estimation of stresses in the other two components.

## 5. Discussion   

Although ND strain measurement is a well established technique, there are still a few key issues that might affect its reliability. One of them is the accurate measurement of the stress-free lattice parameter (*a*_0_) for the evaluation of strain (Withers *et al.*, 2007 ▸). The change in the stress-free lattice parameter due to a non-uniform thermal history and microstructural changes such as compositional discontinuities can result in measurement of pseudo strains. Therefore, the measurement of sufficient stress-free samples from representative locations in the weldment is required (Krawitz & Winholtz, 1994 ▸). In the past, research was conducted on the development of standards for RS measurement using ND, also suggesting an optimal practice for stress-free lattice parameter measurements (Webster *et al.*, 2002 ▸). Several geometries have been defined for the extraction of the stress-free samples, including combs, cylindrical pins and cubes (Hughes *et al.*, 2003 ▸; Daymond & Johnson, 2001 ▸).

The measurement gauge volume is defined by the incident neutron beam and collimator dimensions. The nominal gauge volume in the diffractometer is considered to be cuboid (can also be parallelepiped) with its centroid defined at the intersection of the incident and diffracted neutron beams. However, the effective gauge volume is instrument dependent (Suzuki *et al.*, 2013 ▸; Silvani *et al.*, 2005 ▸), so the measurement is conducted using a sampling gauge volume that is part of the instrumental gauge volume. Texture and neutron beam absorption can have a significant effect on the geometric location and centroid of the sampling gauge volume within the sample (Hutchings *et al.*, 2005 ▸; Price *et al.*, 2008 ▸). In a non-absorbing material this would be the same as the centroid of the instrumental gauge volume. However, attenuation of neutrons within the sample and/or partial burial of the sampling volume could shift it (Wang *et al.*, 1998 ▸; Hsu *et al.*, 1995 ▸). The latter was also studied using the TG6 sample, varying also the gauge area diagonal dimension. Wimpory *et al.* (2018 ▸) showed that the effective measuring position is displaced from the translator position of the diffractometer. By measuring the same locations on the sample twice, first from the top and then from the bottom surface of the sample, they were able to quantify the shift in the measured profile as a function of the gauge volume. The measurement of the same location more than once and at different angles was also proposed to overcome the large grain size effects in the fusion zone of welds (Wimpory *et al.*, 2009 ▸).

Non-uniform plastic deformation in welding could cause the development of large inter-granular residual strains (Hutchings *et al.*, 2005 ▸). The grain size could also contribute to uncertainties in the ND measurements. The larger the grains, the fewer grains are diffracting. The counting statistics also depend on the gauge volume (Holden *et al.*, 2015 ▸; Wimpory *et al.*, 2010 ▸). This could be overcome by rocking the sample, thus allowing more grains to be diffracting within a given gauge volume (Neov *et al.*, 2008 ▸).

**Figure 11:**
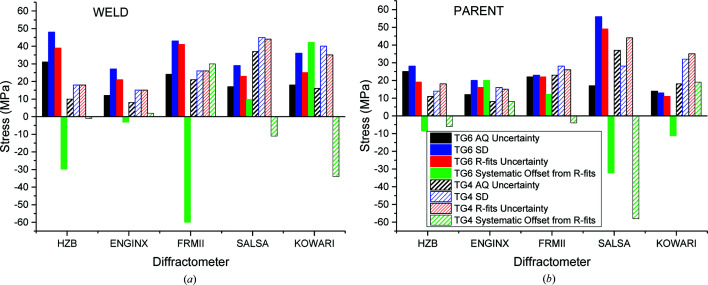
Analysis of quoted and inferred uncertainties for the BD line locations measured in (*a*) the weld and (*b*) the parent region only on different instruments for the TG6 and TG4 specimens.

**Figure 12:**
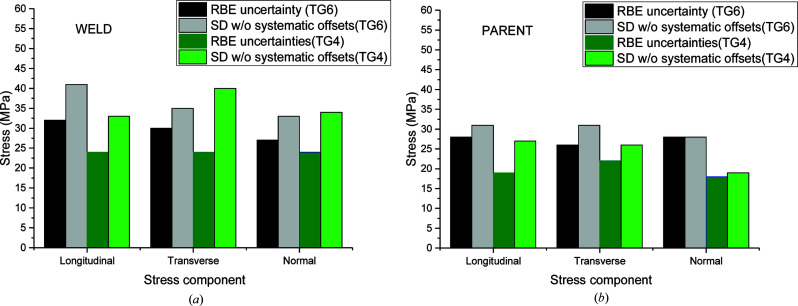
Analysis of inferred uncertainties for the three orthogonal stress components over all data sets of the instruments for the BD line locations measured in (*a*) the weld and (*b*) the parent region only for the TG6 and TG4 specimens.

A comparison of the average quoted (AQ) uncertainties (*i.e.* associated with the fitting of Bragg peaks only), the conventional standard deviation (SD) and the *R*-fits approach of the residuals after subtracting the mean for the TG4 and TG6 data is presented in Fig. 11 ▸. The uncertainties, presented separately for the parent and weld regions, are instrument dependent and were inferred after analysing BD line data only. The systematic error values are in general larger in the weld than in the parent region due to the large grains in the fusion zone that affect the counting statistics, as discussed previously. The performed analysis also allowed a global approach covering all data rather than individual data sets. The RBE actual uncertainties and the SD after removing systematic offsets were calculated for each stress component for both the TG4 and TG6 benchmarks. The same procedure was followed for the classification of the data sets and the uncertainties are presented in Fig. 12 ▸. The Bayesian analysis tends to give a lower bound of uncertainty estimation, whereas the SD tends to give an upper bound and the actual values are somewhere in between. The uncertainty is higher in weld metal due to grain size statistics. The dilution confirmed for the TG6 specimen is also expected to raise the uncertainty.

The uncertainties on different instruments look fairly similar for the parent and weld materials. The uncertainty in the measurements on ENGIN-X is generally lower, possibly due to fitting of multiple peaks rather than one, which mitigates potential grain size issues. To compare the two materials one needs to bear in mind the TG4/TG6 ratio of *E* values used for the estimation of the stresses. For single peak fitting, the ratio of crystallographic moduli was 183.6/206 GPa, whereas the ratio of bulk *E* used on ENGIN-X was 194.7/206 GPa.

The RBE uncertainties for the weld are ∼30 MPa and drop only to ∼25 MPa in the region of parent material. The SD uncertainties after removing the systematic offsets are slightly higher and within a 30–40 MPa range. The RBE uncertainties assessed for the TG4 benchmark using data measured on the same instruments are lower by only 5–10 MPa. This is an outstanding outcome considering that the TG6 specimen was much more challenging to measure.

## 6. Conclusions   

A number of neutron diffraction RS measurements have been performed on the NeT TG6 specimen using neutrons from reactor and spallation sources. Three orthogonal stress components were determined along several lines in the same plate. Measurements were also conducted on stress-free samples from representative locations in a cut specimen. Data were analysed using information from macroscopic characterization studies. The following conclusions were drawn:

(i) A significant variation was revealed between the weld and parent lattice spacing in the unstrained condition. The lattice spacing also varies between the different weld beads due to differences in chemical composition caused by dilution with parent material. A chemical composition analysis was conducted by EPMA and revealed a significant difference in Fe, Cr and other alloying elements.

(ii) A position-fitting approach adopted to estimate the stress-free lattice parameters works well. Here, the cross-sectional profiles of the transverse plane B at weld mid-length and of the longitudinal plane D on the weld centre line were superimposed on metallography of the fusion zone profiles in the transverse and longitudinal directions, respectively. This enabled accurate estimation of parent and weld fractions within the gauge volume at locations close to the fusion boundary, thereby allowing the calculation of stress-free lattice parameters from the fractions of parent and weld in the gauge volume by applying a linear rule of mixtures to the stress-free lattice parameter for each constituent.

(iii) The zero normal stress approach appears to produce reasonable stress results in this relatively thin plate, but caution must be taken when analysing thicker plates, where the zero normal stress assumption does not apply.

(iv) Five measurement data sets obtained from the same specimen on different instruments have been used for the calculation of a robust Bayesian estimate (RBE) of the mean using the ‘duff data’ approach for each stress component and for all measured lines. This method is less susceptible to outliers and allowed a more reliable judgement of RS distribution in the component.

(v) The RBE uncertainties have been calculated for each stress component individually, and also separately for points in the weld and parent material. They were inferred using all data sets acquired along the BD line only, by subtracting the systematic uncertainty from each individual set. The procedure was performed for both TG6 and TG4 benchmarks using data sets acquired on the same diffractometers and fitted using the *d*_0_ stress-free samples (not the KOWARI set).

(vi) The average quoted uncertainties, based strictly on the counting statistics and ignoring other sources of error like chemical composition gradients, sample to stress-free sample variability or property anisotropy, are significantly lower than the actual uncertainties. The systematic offsets were in general higher in the weld due to the lower counting statistics. The uncertainties in both cases do not seem to be dependent on the stress component.

(vii) The actual RBE uncertainties for the measurements on the TG6 specimen were found to be about 30 MPa for weld and 25 MPa for parent material. The uncertainties estimated for the TG4 benchmark were 25 and 20 MPa, respectively. This is an outstanding outcome considering that the TG6 specimen was much more challenging in terms of both measurements and data analysis.

It should be noted that this study examined two welds made in a controlled environment and measurements were performed by the participants following instructions in a protocol. This study also serves as a more statistically robust estimation of the typical uncertainties associated with RS measurements in welds using the neutron diffraction technique.

## Figures and Tables

**Table:** The quantities stated are from the mill certificates acquired upon the materials’ procurement. The mill certificates conformed to the EN10204 standard.

Material	C	Si	Mn	Cr	Ni	S	Nb	Ti	Fe
Alloy 600 (wt%)	0.07	0.12	0.48	15.54	74.35	0.001	0.10	0.006	9.33
Alloy 82 (wt%)	0.009	0.08	3.25	20.8	72.7	0.001	2.6	0.319	0.59

**Table:** 

Material	Yield 0.2% (MPa)	Ultimate tensile stress (MPa)	Elongation (%)
Alloy 600	401	706	40.4
Alloy 82	380	620	35

**Table 2:** The line measurements performed on each instrument, along with the method adopted in each case to calculate the stresses

Instrument	Source type	Measured line(s)	Stress calculation method
ENGIN-X, ISIS (UK)	Pulsed	BD	*d*_0_ pin
		B2	Position fitting
		D2	Position fitting
SALSA, ILL (France)	Reactor	BD	*d*_0_ pin
		B2	Zero normal stress
		D2	Zero normal stress
STRESS-SPEC, FRM II (Germany)	Reactor	BD	*d*_0_ pin
		B2	Zero normal stress
		D2	Zero normal stress
E3, HZB (Germany)	Reactor	BD	*d*_0_ pin
KOWARI, ANSTO (Australia)	Reactor	BD	Zero normal stress
		B2	Zero normal stress
		D2	Zero normal stress
